# Determination of the Elasticity Modulus of 3D-Printed Octet-Truss Structures for Use in Porous Prosthesis Implants

**DOI:** 10.3390/ma11122420

**Published:** 2018-11-29

**Authors:** Ali Bagheri, Irene Buj-Corral, Miquel Ferrer Ballester, Maria Magdalena Pastor, Francesc Roure Fernandez

**Affiliations:** Escola Tècnica Superior d’Enginyeria Industrial de Barcelona (ETSEIB), Avinguda Diagonal, 647, 08028 Barcelona, Spain; irene.buj@upc.edu (I.B.-C.); miquel.ferrer@upc.edu (M.F.B.); m.magdalena.pastor@upc.edu (M.M.P.); francesc.roure@upc.edu (F.R.F.)

**Keywords:** modulus of elasticity, octet truss, 3D printing, scaffold, compressive strength

## Abstract

In tissue engineering, scaffolds can be obtained by means of 3D printing. Different structures are used in order to reduce the stiffness of the solid material. The present article analyzes the mechanical behavior of octet-truss microstructures. Three different octet structures with strut radii of 0.4, 0.5, and 0.6 mm were studied. The theoretical relative densities corresponding to these structures were 34.7%, 48.3%, and 61.8%, respectively. Two different values for the ratio of height (H) to width (W) were considered, H/W = 2 and H/W = 4. Several specimens of each structure were printed, which had the shape of a square base prism. Compression tests were performed and the elasticity modulus (E) of the octet-truss lattice-structured material was determined, both, experimentally and by means of Finite Element Methods (FEM). The greater the strut radius, the higher the modulus of elasticity and the compressive strength. Better agreement was found between the experimental and the simulated modulus of elasticity results for H/W = 4 than for H/W = 2. The octet-truss lattice can be considered to be a promising structure for printing in the field of tissue engineering.

## 1. Introduction

In tissue engineering, different structures or scaffolds, which reproduce the shape of tissues, are obtained [1]. The design of the architecture of the scaffolds at a macro, micro, and nano level, is essential for the structure, nutrient transport, and cell-matrix interaction conditions [2]. Scaffolds must fill the defect cavities and they have to be biocompatible. They require a certain surface morphology to enhance cell attachment and proliferation. They need to be sufficiently strong and have similar stiffness to that of the surrounding tissues. They also require appropriate porosity, pore size, and ratio of surface to volume, to assure permeability [3,4]. The modulus of elasticity of the bulk material can be reduced by the use of porous structures of the same material. A network structure is a microstructure that is formed by the cells of a block. The lattice materials are categorized as Stretching Dominated Lattice Material (SDLM) and Bending Dominated Lattice Materials (BDLM).

In the case of the SDLM, a much stiffer and resistant material per unit mass can be provided by governing the nodal connectivity. This material crumbles with the stretching of its struts. The microscopic structure analysis showed that there is a direct relationship between their stiffness and resistance values, due to an increase in the density ratio [5]. In addition, the lower-density network materials undergo failure with plastic bending, even if the macroscopic load is imposed under a uniaxial tensile load [6]. The BDLM materials are composed of struts connected at nodes. Its main characteristic is the low-joint connectivity (the number of struts that meet in the same node).

In recent years, 3D printing has eased the production of microstructural elements in complex shapes [7]. Wettergreen used the Finite Element Analysis (FEA) to estimate microstructural network properties [8]. Williams et al. [9] found that the experimental modulus of the elasticity for Polycaprolactone (PCL) grid networks produced by Selective Laser Sintering (SLS), was higher than the one obtained by the Finite Element Methods (FEM). This was attributed to the differences in packing of pores and in strut sizes. Luxner et al. [10] studied different structures, such as the Simple Cubic (SC), Gibson Ashby (GA), Body Centered Cubic (BCC), and the Reinforced Body Centered Cubic (RBCC). Both Digital Light Processing (DLP) and SLS were used in these experimental tests. Similar values between experimental and FEM tests were reported for all the structures, except for the SC. This was attributed to the high directional sensitivity of the SC structure. Studies have been performed on the additively-manufactured cubic scaffolds composed of several unit cells of Schoen’s F-RD surface, a central chamber with tubes to the alternating corners of a cube (bending dominated) and Schoen’s I-WP surface, a central chamber with tubes to the corners of a cube (stretching dominated) architectures. The possibility of modifying the mechanical properties by controlling, both, the pore architecture and the relative density of the structures, has been demonstrated [11]. Fernandez et al. investigated three different structures—rectilinear, honeycomb, and line—with different densities and different infill percentages, using the Fused Deposition Modelling (FDM) technology. The rectilinear pattern with a 100% infill had the highest tensile strength of 36.4 MPa [12]. The experimental tests by Ahmed et al. showed that the average modulus of elasticity of the 3D-printed Acrylonitrile Butadiene Styrene (ABS) specimens was lower than that of the regular plastic ABS [13]. A study of different PLA-printed scaffolds showed that the change in mechanical strength was slight and the surface area improved significantly by reducing the scaffolds’ cell size [14]. Arabnejad et al. have shown that the stretch-dominated cell topologies can be used in load-bearing orthopedic applications for bone replacement and that the octet-truss structure promoted bone ingrowth in a canine model [15]. In the same line, Egan et al. tested four types of scaffolds, taking into account the modulus of elasticity and the surface-volume ratio. The scaffolds with the beam-based unit cells showed higher elastic moduli, while the truss-based scaffold tended to attain a greater surface-volume ratio [16]. Hollister et al. stated that the higher the density of the scaffolds, the normalized elastic modulus is higher and the permeability is lower. Different scaffold microstructures also showed different stiffness [17]. The open-porous titanium scaffolds presented a good agreement for cell growth of a sheep’s bone, in vivo. In addition, a higher amount of the newly-formed bone was found surrounding the less-stiff implants [18].

As for the octet-truss structure, recent studies by O’Masta [19] have shown that its cellular structures, such as the Ti-6Al-4V alloy octet lattice, have higher compressive strengths (between 20 and 70 MPa) and higher elastic moduli, when creating low-density metallic trusses. Bonatti and Mohr studied four different structures—solid octet truss (SOT), hollow sphere assembly (HAS), hollow octet truss (HOT), and hybrid truss-sphere assembly (HTS). They found the lowest modulus of 7.1 GPa, for the SOT structures, and the highest modulus of 10.4 GPa, for the HTS structure. In addition, the experimental results were lower than the FEA-simulated results, for all the structures [20]. However, changes in the density of structures can present a sudden drop in mechanical properties [21]. The mechanical properties of the structures can be influenced by several factors, such as the cell size, the length of the struts, the radius of struts, and the degree of mutuality of the octet lattice [22].

In the present paper, the effect of the struts radius on the structure stiffness has been assessed. The structures density was measured and compression tests were carried out. Prismatic samples were printed with a width to height ratio W/H of 2 and 4. FEM analysis was performed and compared to the experimental results. 

## 2. Materials and Methods

**Figure 1:**
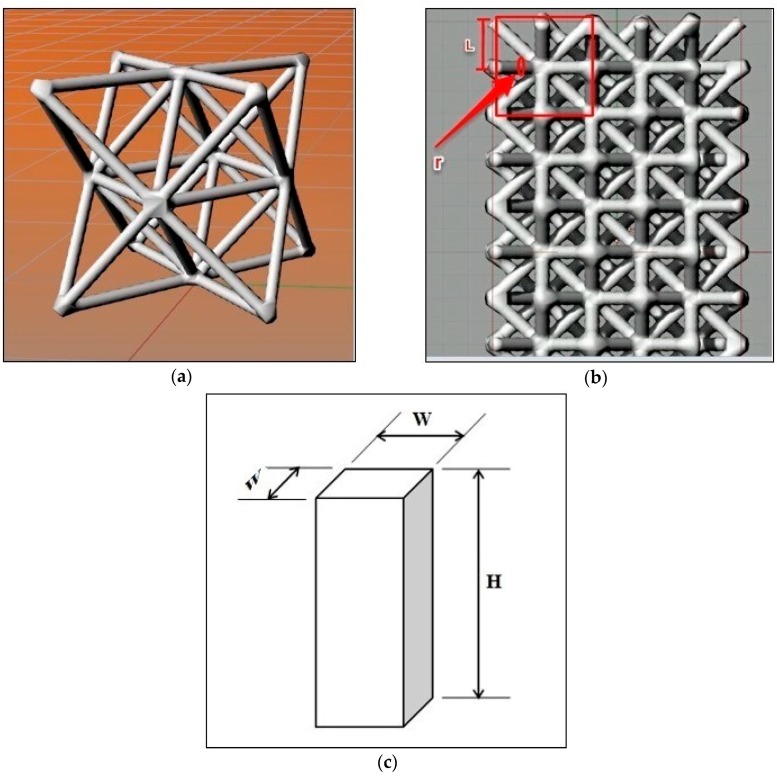
(**a**) Octet-truss structure generated by the Grasshopper; (**b**) Octet-truss with a toggle-true feature (which makes the outer edges of the structure unjointed); (**c**) Prismatic sample for the compression test.

In the octet-truss model, the regular octahedral unit cell core is surrounded by eight tetrahedrons, distributed on its eight faces (Figure 1a). The cell has the shape of a Face Centered Cube (FCC), along with a lattice structure, which has cubic symmetry and isotropic properties [23]. The nodes in this structure are connected to twelve cell elements, in the same manner. In the related literature, the octet-truss network structure is conducted with a solid circular cross-section of struts, especially in the lattice structures, whose cell topology is integrated for constructing an open-cell foam [24]. In the present paper, circular cross-section of struts has also been used.

### 2.1. Printing Tests

Printing tests were performed by means of the Fused Filament Fabrication (FFF) technology, with a Sigma printer, from BCN3D. 

In the present work, Polylactic acid (PLA) was used, which is a synthetic polymer that does not produce toxic fumes when printing. In the literature, PLA, PLA/calcium phosphate glass and chitosan have been used for manufacturing scaffolds that help in tissue repair and regeneration [25]. PLA was also employed for simulating the trabecular structures of the bone tissue [26].

For designing the structure, the Grasshopper plug-in [27] and the Rhinoceros 3D software [28] were used together. The Grasshopper allowed the pieces to be created, through Rhinoceros, with random patterns and forms that could not be created using other drawing software. Two structures were created; each of them were studied separately. The structures were created by Grasshopper, with a cell size of 4 mm and in the toggle graphics mode.

As seen in Figure 1b, the length of each member of the struts is equal to $L\sqrt{2}$, where L is half the length of the cell size (Equation (1)):

(1) $$L\;=\;\frac{Length\;of\;cell}{2}$$

In this structure, the cell is a cube with a side of 4 mm, which encloses the octal structure. Octet-truss structures were designed within a square base prism, with length to width ratios of H/W = 2 and H/W = 4 (Figure 1c). A toggle-false feature was selected so that the program could make the surfaces of the structure, continuous, in the prism, and connected to each other. Preliminary tests showed a higher strength for those prismatic structures that had toggle-false features than those with toggle-true. Therefore, the toggle-false structures have been used in this paper.

After creating the octet structure, it was saved as a stereolithographic (STL) file. Moreover, in order to import this structure into a printer, the file had to be stored in the G-code, in laminate programs, such as Cura Ultimaker Ver. 2.7 [29], used to prepare an appropriate file for printing. The printer is able to read the file as a G-code. The point to be noted is that the printing parameters should also be included in the STL file, prior to saving as a g-code file. The parameters—nozzle size, layer height, infill, and print speed, were selected, with values of 0.4 mm, 0.1 mm, 95% and 40 mm/s, respectively. The other main parameters selected for this work were—printing temperature, initial layer thickness, infill speed, and shell thickness, with values of 200 °C, 100%, 60 mm/s and 0.8 mm, respectively.

Each specimen was manufactured in accordance with Section 6.2 of the ASTM D695-02a standard [30]. It was prismatic in shape, with its length being twice its principal width. Initially, the dimensions were 10 × 10 × 20 mm^3^. However, when the elastic modulus is desired, the test specimen should be of such dimensions that the slenderness ratio is in the range from 11 to 16. In this case, the preferred specimen sizes were 10 × 10 × 40 mm^3^ (Figure 1c and Equations (2)–(5)).

(2) $$\lambda \;=\;\frac{H}{i}$$

where *λ* is the slenderness ratio, *H* is the specimen height, and *i* is the radius of gyration:

(3) $$i\;=\;\sqrt{\frac{I}{A}}\;=\;\sqrt{\frac{\frac{1}{12}W^{4}}{W^{2}}}\;=\;\frac{W}{\sqrt{12}}$$

where *i* is the second moment of area, *A* is the area of the cross-section, and *W* is the width of specimen (length of each side of the cross-sectional area).

For H/W = 2:

(4) $$\lambda \;=\;\frac{20\;\mathrm{mm}}{\left(\frac{10\;\mathrm{mm}}{\sqrt{12}}\right)}≅\;7$$

For H/W = 4 specimens:

(5) $$\lambda \;=\;\frac{40\;\mathrm{mm}}{\left(\frac{10\;\mathrm{mm}}{\sqrt{12}}\right)}≅\;14\in \left[11,\;16\right]$$

Thus, ratio H/W = 4 complies with the specifications of the standard.

### 2.2. Determination of Density

The theoretical density of the octet structure was calculated from the following formulae (Equations (6) and (7)):

(6) $$\bar{\rho }\;=\;\frac{6\sqrt{2}A}{l^{2}}$$

(7) ρ¯ = 62Al2 − c (rl)3

where $\bar{\rho }$ is the ratio of the lattice material density to the solid material density [31], *A* is the cross-section area of each strut, l is the length of each strut, and r is the strut radius. In addition, *c* depends on the detailed geometry of the nodes.

The first formula (Equation (6)) was used for struts with a small radius. This formula is a first-order approximation and gives relatively higher results than the second one (Equation (7)), since it calculates the volume of the node, twice. In the second formula (the high-order approximation of Equation (6)), the *c* value should be calculated according to the regular shape of the nodes. In this study, the nodes could not be drawn with a spherical shape; the shape of the intersection among the struts was used instead. Due to the irregular structure, the Rhinoceros software [28] was used to obtain the density ratio, instead of Equations (6) and (7). First, a 4 × 4 × 4 mm^3^ cube was designed, using this software, and then the characteristics of the PLA were entered into the program. The unit-cell of the octet structure was also designed as a 4 × 4 × 4 mm^3^ cube, with the same material properties. Then, dividing the density of the octet structure (ρ) by the density of the solid in the same cell size ($\rho^{*})$, according to Equation (8), the density ratio was obtained for each of the three different radii 0.4, 0.5 and 0.6 mm.

(8) ρ¯=ρρ*

The specimens were weighed after printing and the experimental density was calculated, based on the actual volume and mass. Three samples of each condition were used for calculating the experimental density.

### 2.3. Compression Tests

**Figure 2:**
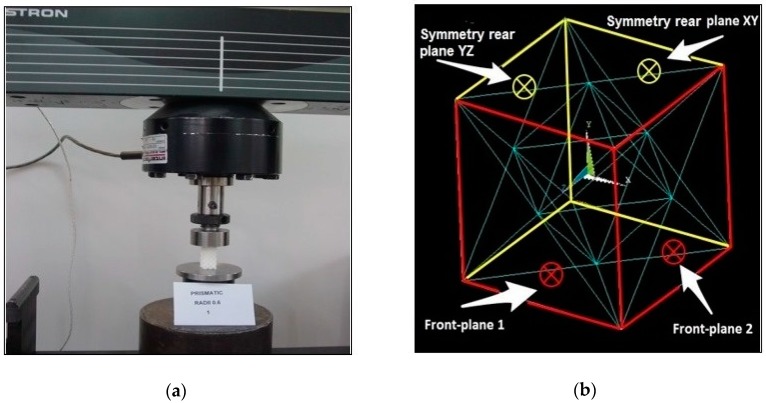
(**a**) A view of the compression test on a toggle-false sample with radii of struts 0.6 mm; (**b**) The structure of the octet-truss modelled with Ansys.

The compression tests of the specimens were performed on an Instron 3366 universal testing machine (Figure 2a), according to the ASTM D695-02a standard protocol. This standard describes the methodology to be followed in determining the mechanical properties of rigid plastics under compression. Three prismatic specimens of 10 × 10 × 20 mm^3^ were printed for each condition (H/W = 2). Five prismatic specimens of 10 × 10 × 40 mm^3^ were printed for each condition (H/W = 4). Both types of specimen were tested. The results are shown for comparison. The test speed was 1.3 ± 0.3 mm/min, which is the test speed given by the standard. Figure 2a shows an example of a compression test.

### 2.4. FEM Analysis

An academic version of the ANSYS v15 FEM code [32] was used for the FEM simulation. In order to simulate the deformation of the octet-truss structure, it was first drawn through the coordinates of its key corners. Each one of the octahedrons and the tetrahedrons were placed within a given 4 mm-sided cube (Figure 2b). The stiffness of the octet depends significantly on the size of this cubic pattern. Three radii *r* of the circular cross-section were analyzed: 0.4, 0.5, and 0.6 mm. The struts lying on the outer faces of the unit-cell were cut in half, due to the symmetry boundary condition. The ten-noded solid element (Solid 187 in Ansys) (Canonsburg, Pennsylvania, United States) was used to mesh the truss members of the lattice structure. This finite element model was then subjected to the following uniaxial compressive boundary conditions (Figure 2b).

The nodes at the bottom face of the octet-truss structure were fixed in the Y direction (Y-symmetry condition). All nodes lying on the top plane were coupled with each other, in the Y direction, i.e., all nodes were forced to share the same UY displacement. In this way, the uniaxial load could be easily applied through the “master node” and the nodes were kept as a horizontal flat surface. The nodes on the XY and YZ rear planes were fixed, in terms of the Z and X directions, respectively, because of the symmetry condition. Likewise, the nodes lying on the XY and YZ front-planes 1 and 2 were coupled with regards to the X and Z directions respectively, in order to keep them as vertical flat surfaces (symmetry conditions). These boundary conditions were verified by the displacement results in Figure 2b. A linear structural analysis was performed for this simulation.

By prescribing a vertical displacement ($\Delta l_{s}$ (of −0.5 mm on the master node in the negative Y direction, the full reaction force (*F*) was obtained, in this single node, as a computational result. Then, the longitudinal Young’s modulus (*E*) could be determined for every radius *r* from Equation (9):

(9) E = σε = FAΔlsls

where σ is the normal Y engineering stress, i.e., the vertical reaction force *F* divided by the initial area *A* of the upper surface of the cubic cell. ε is the longitudinal Y strain, i.e., the prescribed displacement of the upper surface in the Y direction ($\Delta l_{s}$) divided by the initial length of the cell ($l_{s}$).

## 3. Results

### 3.1. Density

The theoretical and experimental densities of the samples are shown in Table 1. As can be seen, similar values were obtained for both theoretical and experimental densities.

The bigger the strut radius, higher the theoretical and experimental relative densities of the prisms. The average experimental density values were similar to the theoretical density values. 

### 3.2. Compression Tests

**Figure 3:**
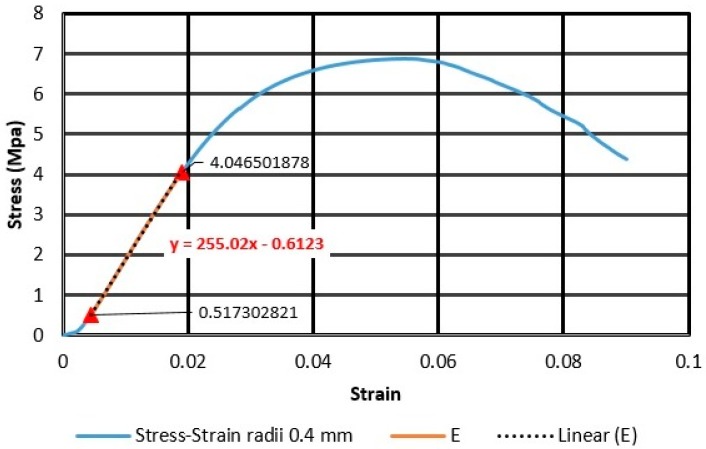
Stress–strain diagram and linear regression equations for the first specimen, with radius 0.4 mm.

Figure 3 shows an example of the linear regression at the initial slope of the stress–strain curve, to obtain the modulus of elasticity.

**Figure 4:**
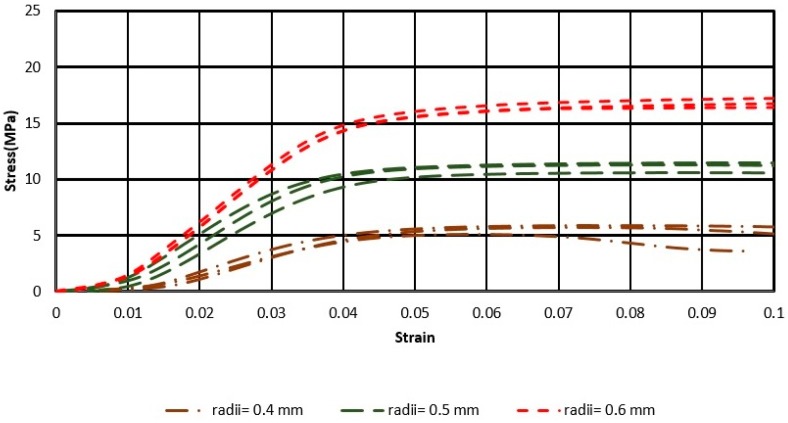
Stress–Strain diagram for the three samples with height-to-width ratio (H/W) = 2, of octet, with radii of struts 0.4, 0.5, and 0.6 mm.

**Figure 5:**
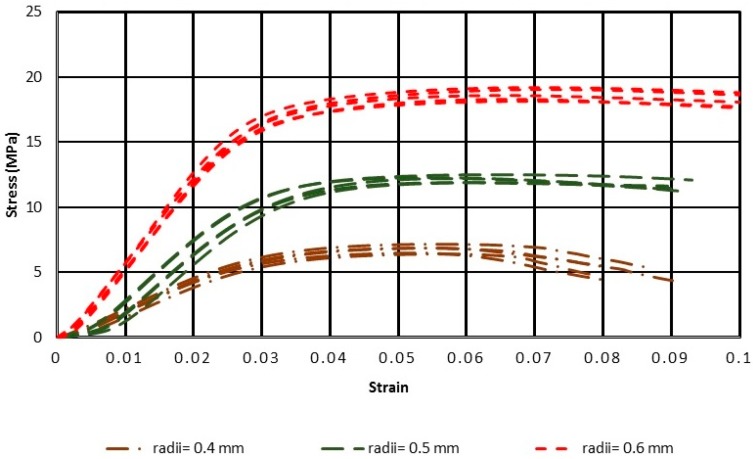
Stress–Strain diagram for the five samples with H/W = 4, of octet, with radii of struts 0.4, 0.5, and 0.6 mm.

The results of the uniaxial compressive tests are presented in Figure 4 and Figure 5, for ratios H/W = 2 and H/W = 4, respectively.

The stress–strain curves showed that the greater was the radius of the struts (r), the higher were the curves and higher was the linear elastic modulus. On the other hand, the stress–strain curves of the specimens with H/W = 4, were higher than those of the specimens with H/W = 2. 

According to Figure 4 and Figure 5, if the same radius of the struts is considered, compressive strength for samples with H/W = 4 is higher than that for H/W = 2. In addition, the curves obtained showed that the slope of the stress–strain curves for the longer samples was higher than that for the shorter ones; samples with a greater length, met the elastic stage sooner. 

The regression equations of the five samples for each structure with H/W = 4 and the three samples with H/W = 2 are presented in Table 2 and Table 3, respectively.

For each experiment, the mean value of the modulus of elasticity was calculated. The slope of the regression equations corresponded to the modulus of elasticity. The average values are shown in Table 4.

The greater was the radius of the struts, the higher was the average equivalent Young’s modulus; as was expected. Lower Young’s modulus and higher standard deviation values of the Young’s modulus, were found for H/W = 2 than for H/W = 4.

Additionally, the ultimate compressive strength (UCS) obtained from the experimental results is shown in Table 5. The greater the radius of the struts, the higher was the UCS. Regarding the average values observed, the compressive strength increased along with an increase in the radius of the struts. It was also found that by increasing the radius by 0.1 mm, the compressive strength of the structure was, approximately, doubled, due to the increase in density from 42.1(%) to 57.1(%). Moreover, it could be concluded that the compressive strength for the specimens with H/W = 4 was higher than that for specimens having an H/W = 2. Standard deviation values ranged from 0.252 to 0.468.

### 3.3. FEM Results

Linear elastic simulations were performed on the above described single-cell FEM models. The full-solid PLA properties were used. Although the elastic modulus value for the commercial amorphous PLA, commonly found in the literature, is 3500 MPa [33], other values have also been reported, within a certain range. In this study, the reference values chosen were E = 2500 MPa and ν = 0.36, according to the results of preliminary experimental tests with H/W = 2.

**Figure 6:**
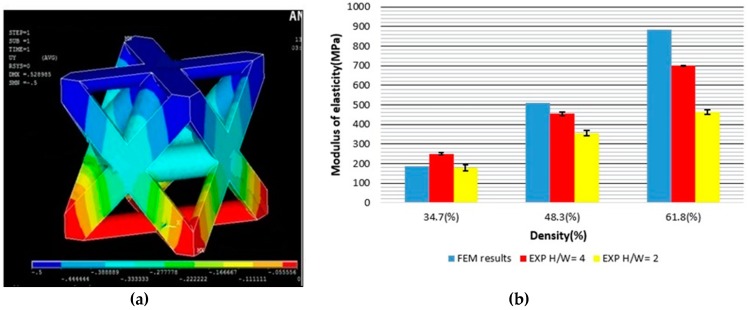
(**a**) UY results of the octet-truss. (**b**) Modulus of elasticity (MPa) vs Density (%) (Comparison of FEM results with experimental results for H/W =2 and H/W=4 respectively).

Figure 6a shows the Y displacement map (UY) of the octet-truss structure.

As shown in Figure 6b, the experimental results for a radius of 0.4 mm were very similar to the simulated ones. For radii 0.5 and 0.6 mm, the experimental values were lower than the simulated ones. The results of H/W = 4 matched the simulation, better, than the results of H/W = 2. This agreed with the recommendation of the standard—increasing the sample height to prevent the effect of cross-section deformation, due to the compression test. Furthermore, a higher variability was found for H/W = 2 than for H/W = 4.

In Table 6, the equivalent Young’s modulus, obtained from the FEM calculations, for each radius value, is presented.

The greater the radius of struts, the higher is the obtained equivalent Young’s modulus.

**Figure 7:**
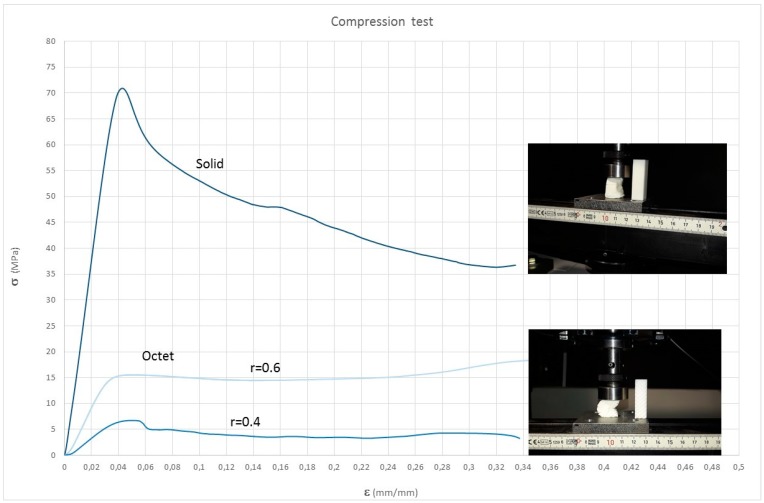
Stress–strain curves of the octet-truss structures and the solid-printed structures, up to the final densification.

As an illustration of the specimen densification process, the stress–strain curves are presented in Figure 7. These were obtained from a compression test, until extreme deformation of the specimens occurred. Three curves are shown—a solid specimen and two octet specimens—with different radii of the struts. It should be noted that the specimens have been manufactured under conditions different from the previous ones, so the numerical values could not be compared.

The curves of the octet structures show a linear elastic area, followed by a plateau, while the curves of the solid structure shows a descending curve, after the linear elastic area.

## 4. Discussion

In order to replace natural human bones with implants, surgeons assess different criteria. Modulus of elasticity is one of the main items for the selection of material and structure. In addition, the modulus of scaffolds can vary with time because of the possible cell infiltration and extracellular matrix (ECM) deposition [34]. Analytical and clinical studies are required to determine the modulus of elasticity that will result in the best patient outcomes [35].

Some studies show that, for bone growth and interconnectivity of the cellular structures, the octet-truss has better results, compared to other porous structures, and it is reconfigurable to match the bone elasticity [36]. This fact has also been observed in the present study. Junchao et al. worked on a simplified porous structure, which proved that the ultimate strength and elasticity modulus have a nearly linear increase with the growth of the struts [37]. The type of the unit cell is a key factor for the biological and mechanical properties of the porous structures, with regards to the bone tissue regeneration [38]. With the help of the compression test, the compressive strength and elastic modulus of the printed structures can be assessed so that they match the requirements of different structures [39]. The study of the bone matrix showed that the increase or decrease in bone mass were related to its stress–strain state [40].

Different plastic materials have been used for tissue regeneration [41]. For example, poly(d,l-lactic-co-glycolic acid) (PLGA) and bothpoly(ε-caprolactone) (PCL) are used in bone tissue regeneration [42]. PLGA provided a faster polymer degradation rate than the PCL. The decrease of the molecular weight for PLGA was about 50%, in vitro, and 70%, in vivo, after 28 days, while for the PCL it was about 30%, in vitro, and 40%, in vivo, after the same time [43]. In the present study, PLA was used, which is more stable than the PLGA and the PCL [44]. On the other hand, mechanical properties of the PLA are suitable for application in bone tissue engineering [45]. The PLGA, however, needs to be combined with other polymers to improve its relatively poor mechanical properties (especially its Young’s modulus) [46]. Another important advantage of these materials is that they have been approved for clinical use [47]. 

Along with polymers, other types of materials are used in tissue engineering. Metals can be employed for manufacturing scaffolds because of its high strength and safety, for use in vivo. However, they also have some disadvantages, such as the limited availability of printing technology and the toxicity of metals when released to the body. Different metals have been printed, for example, stainless steel, cobalt–chromium alloys, titanium alloys, nitinol, etc. [41]. Tantalum has also been used for the same purpose. For example, the Young’s modulus of the porous Ta structures is low, ranging from 1.5 to 20 GPa [47], and can be used in hip and knee-joint reconstruction [48]. Metal matrix syntactic foams (MMSFs) are closed-cell porous structures with high stiffness-to-weight ratio. For example, Katona et al. reported modulus of elasticity values around 20 GPa for two different types of aluminium foams [49]. Linul et al. tested stiffness of aluminium foam panels with stainless steel reinforcement, at extreme temperatures, between −196 °C and 250 °C. They observed that, the higher the temperature, the lower the stiffness. All stress–strain graphs showed a short elastic area, followed by a long plateau and a final densification area. At 25 °C, they reported modulus of elasticity values up to 336 MPa, with longitudinal reinforced foams [50].

As for ceramics, they show high mechanical strength and biocompatibility [41]. In addition, some ceramics, like hydroxyapatite (HA) show properties similar to those of natural bones [51] and can be used for bone regeneration. Leukers et al. printed HA scaffolds with internal surfaces with a 45° inclination to enhance cell proliferation [52]. Calcium silicate has also been employed to print scaffolds with high bone-healing properties [53]. However, printing technologies for ceramics are less developed than the printing technologies for polymers. As for modulus of elasticity of biomaterials, modulus of elasticity of ceramics is generally higher than the modulus of metals, and this is higher than the modulus of polymers [54]. In addition, the stress–strain curve for ceramics only shows the elastic zone, while for metals, the elastic zone, the plastic zone, and necking are shown. For polymers, the curves show, both, elastic and plastic zone.

In this study, the radius of the struts was varied, in order to investigate their effect on the modulus of elasticity of the structure.

## 5. Conclusions

In this study, square base prisms were printed with an octet-truss structure. The density of specimens was calculated. Compression tests were carried out and the experimental modulus of elasticity of the octet structure was compared to the results from the FEM calculations. The main conclusions are as follows.

The FEM is a useful tool for predicting the behavior of printed octet structure with a low strut radius, when the structure deforms mainly by stretching. For a greater strut radius, the experimental results of the Young’s modulus were lower than that predicted by FEM, probably because of bending.

Both FEM and the experimental results showed that increasing the radius of the struts in the PLA structures, increases the stiffness and, therefore, the modulus of elasticity of the structures. The compression strength also increases with the radius of the struts. The results for H/W = 4 were more similar to the simulated ones, than the results for H/W = 2. Thus, the results agree with the recommendation of the standard ASTM D695-02a, of using H/W = 4, when determining the modulus of elasticity.

Due to its mechanical properties, this structure can be used for printing structures for tissue regeneration. Mechanical properties, such as elasticity modulus can be varied from the selection of the different radius of the struts. In the future, other geometric variables of the structure, such as cell size or length of struts, can also be varied, in order to obtain different structures.

## Figures and Tables

**Table 1:** Comparison between the theoretical and experimental relative density of the octet structure, for the different radii of the struts.

Reference	Radii of Struts (mm)	Theoretical Relative Density (%)	Experimental Relative Density (%)	Average Experimental Relative Density (%)	Standard Deviation of Experimental Relative Density (%)
[1]	0.4	34.7	34.6	35.2	0.513
			35.3		
			35.6		
[2]	0.5	48.3	49.1	48.0	1.018
			47.8		
			47.1		
[3]	0.6	61.8	58.7	58.8	0.891
			57.9		
			59.7		

**Table 2:** Linear regression equations for H/W = 4.

Reference of Structures	Radius (mm)	Regression Equations
1a	0.4	Y = 255.02X − 0.6123
1b		Y = 248.08X − 0.5865
1c		Y = 247.11X − 0.3492
1d		Y = 247.23X − 0.2129
1e		Y = 256.24X − 0.4689
2a	0.5	Y = 452.26X − 2.7196
2b		Y = 443.74X − 3.3128
2c		Y = 462.55X − 1.8626
2d		Y = 452.26X − 2.7196
2e		Y = 462.81X − 1.8526
3a	0.6	Y = 700.15X − 1.5756
3b		Y = 701.18X − 1.9425
3c		Y = 698.99X − 1.0848
3d		Y = 701.70X − 1.0791
3e		Y = 698.68X − 1.1595

**Table 3:** Linear regression equations for H/W = 2.

Reference	Radius (mm)	Regression Equations
1a	0.4	Y = 162.86X − 1.8463
1b		Y = 186.14X − 2.6123
1c		Y = 191.27X − 2.0990
2a	0.5	Y = 361.74X − 2.2872
2b		Y = 339.97X − 3.3646
2c		Y = 365.58X − 3.0434
3a	0.6	Y = 473.57X − 3.6558
3b		Y = 451.52X − 2.9425
3c		Y = 467.35X − 3.0848

**Table 4:** Young’s modulus equivalent to each structure of the octet-truss, given by the compression test.

Reference	Radius (mm)	Average Equivalent Young’s Modulus (MPa) for H/W = 4	Standard Deviation of Equivalent Young’s Modulus (MPa) for H/W = 4	Average Equivalent Young’s Modulus (MPa) for H/W = 2	Standard Deviation of Equivalent Young’s Modulus (MPa) for H/W = 2
[1]	0.4	250.7	4.504	180.1	15.140
[2]	0.5	454.7	8.053	355.8	13.811
[3]	0.6	700.1	1.320	464.1	11.369

**Table 5:** Ultimate Compressive Strength (UCS) equivalent to each structure of the octet-truss obtained from the compression test.

Reference	Radius (mm)	Compressive Strength (MPa) for H/W = 4	Average Compressive Strength (MPa) for H/W = 4	Standard Deviation of Compressive Strength (MPa) for H/W = 4	Compressive Strength (MPa) For H/W = 2	Average Compressive Strength (MPa) for H/W = 2	Standard Deviation of Compressive Strength (MPa) for H/W = 2
1a	0.4	6.87	6.76	0.318	5.07	5.56	0.430
1b		6.40			5.76		
1c		6.48			5.86		
1d		7.18			-		
1e		6.85					
2a	0.5	11.93	12.17	0.252	11.45	11.12	0.468
2b		11.93			10.59		
2c		12.53			11.34		
2d		12.23			-		
2e		12.25					
3a	0.6	19.03	18.65	0.465	17.13	14.41	0.369
3b		18.57			17.83		
3c		18.32			17.28		
3d		19.21			-		
3e		18.11					

**Table 6:** Equivalent Young’s modulus obtained from the FEM.

Reference of Structure	Radii of Struts (mm)	Equivalent Young’s Modulus (MPa)
1	0.4	184.9
2	0.5	508.6
3	0.6	886.2

## Nomenclature and Terminology

FDM	fused deposition modeling
FFF	fused filament fabrication
PLA	polylactic acid
ABS	acrylonitrile butadiene styrene
W	width of specimen
H	height of specimen
R	radius of struts
l	length of struts
$l_{s}$	initial length of the cell for FEM

## References

[1] An J., Teoh J.E.M., Suntornnond R., Chua C.K. (2015). Design and 3D Printing of Scaffolds and Tissues. Engineering.

[2] Chiulan I., Frone A., Brandabur C., Panaitescu D. (2017). Recent Advances in 3D Printing of Aliphatic Polyesters. Bioengineering.

[3] Tan X.P., Tan Y.J., Chow C.S.L., Tor S.B., Yeong W.Y. (2017). Metallic powder-bed based 3D printing of cellular scaffolds for orthopaedic implants: A state-of-the-art review on manufacturing, topological design, mechanical properties and biocompatibility. Mater. Sci. Eng. C.

[4] Hollister S., Lin C., Saito E., Lin C., Schek R., Taboas J., Williams J., Partee B., Flanagan C., Diggs A. (2005). Engineering craniofacial scaffolds. Orthod. Craniofacial Res..

[5] Ashby M.F. (2005). Materials selection in mechanical design. MRS Bull..

[6] Deshpande V.S., Fleck N.A. (2001). Collapse of truss core sandwich beams in 3-point bending. Int. J. Solids Struct..

[7] Rochus P., Plesseria J.-Y., Van Elsen M., Kruth J.-P., Carrus R., Dormal T. (2007). New applications of rapid prototyping and rapid manufacturing (RP/RM) technologies for space instrumentation. Acta Astronaut..

[8] Wettergreen M.A., Bucklen B.S., Starly B., Yuksel E., Sun W., Liebschner M.A.K. (2005). Creation of a unit block library of architectures for use in assembled scaffold engineering. CAD Comput. Aided Des..

[9] Williams J.M., Adewunmi A., Schek R.M., Flanagan C.L., Krebsbach P.H., Feinberg S.E., Hollister S.J., Das S. (2005). Bone tissue engineering using polycaprolactone scaffolds fabricated via selective laser sintering. Biomaterials.

[10] Luxner M.H., Stampfl J., Pettermann H.E. (2005). Finite element modeling concepts and linear analyses of 3D regular open cell structures. J. Mater. Sci..

[11] Kadkhodapour J., Montazerian H., Darabi A.C., Zargarian A., Schmauder S. (2017). The relationships between deformation mechanisms and mechanical properties of additively manufactured porous biomaterials. J. Mech. Behav. Biomed. Mater..

[12] Fernandez-Vicente M., Calle W., Ferrandiz S., Conejero A. (2016). Effect of Infill Parameters on Tensile Mechanical Behavior in Desktop 3D Printing. 3D Print. Addit. Manuf..

[13] Ahmed M., Islam M., Vanhoose J., Rahman M. (2017). Comparisons of Elasticity Moduli of Different Specimens Made Through Three Dimensional Printing. 3D Print. Addit. Manuf..

[14] Habib F.N., Nikzad M., Masood S.H., Saifullah A.B.M. (2016). Design and Development of Scaffolds for Tissue Engineering Using Three-Dimensional Printing for Bio-Based Applications. 3D Print. Addit. Manuf..

[15] Arabnejad S., Burnett Johnston R., Pura J.A., Singh B., Tanzer M., Pasini D. (2016). High-strength porous biomaterials for bone replacement: A strategy to assess the interplay between cell morphology, mechanical properties, bone ingrowth and manufacturing constraints. Acta Biomater..

[16] Egan P.F., Ferguson S.J., Shea K. (2017). Design of Hierarchical Three-Dimensional Printed Scaffolds Considering Mechanical and Biological Factors for Bone Tissue Engineering. J. Mech. Des..

[17] Hollister S.J. (2005). Porous scaffold design for tissue engineering. Nat. Mater..

[18] Wieding J., Lindner T., Bergschmidt P., Bader R. (2015). Biomechanical stability of novel mechanically adapted open-porous titanium scaffolds in metatarsal bone defects of sheep. Biomaterials.

[19] O’Masta M.R., Dong L., St-Pierre L., Wadley H.N.G., Deshpande V.S. (2017). The fracture toughness of octet-truss lattices. J. Mech. Phys. Solids.

[20] Bonatti C., Mohr D. (2017). Large deformation response of additively-manufactured FCC metamaterials: From octet truss lattices towards continuous shell mesostructures. Int. J. Plast..

[21] Sciencedirect S. (2016). Mechanical evaluation of porous titanium (Ti6Al4V) structures with electron beam melting (EBM). J. Mech. Behav. Biomed. Mater..

[22] Johnston B. (2016). High-Strength Fully Porous Biomaterials for Bone Replacement and Their Application to a Total Hip Replacement. Master’s Thesis.

[23] Renton J.D. (2002). Elastic Beams and Frames.

[24] Challapalli A. (2015). Loading Mode Dependent Effective Properties of Octet-Truss Lattice Structures Using 3D Printing.

[25] Almeida C.R., Serra T., Oliveira M.I., Planell J.A., Barbosa M.A., Navarro M. (2014). Impact of 3-D printed PLA- and chitosan-based scaffolds on human monocyte/macrophage responses: Unraveling the effect of 3-D structures on inflammation. Acta Biomater..

[26] Buj-Corral I., Bagheri A., Petit-Rojo O. (2018). 3D Printing of Porous Scaffolds with Controlled Porosity and Pore Size Values. Materials.

[27] Rutton D. Grasshopper 2007, Grasshopper. http://www.grasshopper3d.com.

[28] Robert McNeel Rhinoceros 3D 5 1980. https://www.rhino3d.com.

[29] Cura 2.7. https://ultimaker.com/en/products/50588-cura-27.

[30] ASTM Standard D1621 (2004). Standard Test Method for Compressive Properties of Rigid Cellular Plastics.

[31] Abdelhamid M., Czekanski A. (2018). On the Effective Properties of the Octet-Truss Lattice. SolMech.

[32] John A. Swanson Ansys 15 1970. http://www.ansys.com/academic/free-studentproducts.

[33] Farah S., Anderson D.G., Langer R. (2016). Physical and mechanical properties of PLA, and their functions in widespread applications—A comprehensive review. Adv. Drug Deliv. Rev..

[34] Goyal R., Guvendiren M., Freeman O., Mao Y., Kohn J. (2017). Optimization of Polymer-ECM Composite Scaffolds for Tissue Engineering: Effect of Cells and Culture Conditions on Polymeric Nanofiber Mats. J. Funct. Biomater..

[35] Heary R.F., Parvathreddy N., Sampath S., Agarwal N. (2017). Elastic modulus in the selection of interbody implants. J. Spine Surg..

[36] Cheng A., Humayun A., Cohen D.J., Boyan B.D., Schwartz Z. (2014). Additively manufactured 3D porous Ti-6Al-4V constructs mimic trabecular bone structure and regulate osteoblast proliferation, differentiation and local factor production in a porosity and surface roughness dependent manner. Biofabrication.

[37] Li J., Zang Y., Wang W. (2016). Elastic Modulus and Stress Analysis of Porous Titanium Parts Fabricated by Selective Laser Melting. J. Harbin Institute Tech..

[38] Zhang X.Y., Fang G., Zhou J. (2017). Additively manufactured scaffolds for bone tissue engineering and the prediction of their mechanical behavior: A review. Materials.

[39] Xu Y., Zhang D., Zhou Y., Wang W., Cao X. (2017). Study on topology optimization design, manufacturability, and performance evaluation of Ti-6Al-4V porous structures fabricated by selective laser melting (SLM). Materials.

[40] Frost H. (1987). The mechanostat: A proposed pathogenic mechanism of osteoporoses and the bone mass effects of mechanical and nonmechanical agents. Bone Min..

[41] Do A.-V., Khorsand B., Geary S.M., Salem A.K. (2015). 3D Printing of Scaffolds for Tissue Regeneration Applications. Adv. Healthc. Mater..

[42] Griffith L.G. (2000). Polymeric biomaterials. Acta Mater..

[43] Sung H.J., Meredith C., Johnson C., Galis Z.S. (2004). The effect of scaffold degradation rate on three-dimensional cell growth and angiogenesis. Biomaterials.

[44] Da Silva D., Kaduri M., Poley M., Adir O., Krinsky N., Shainsky-Roitman J., Schroeder A. (2018). Biocompatibility, biodegradation and excretion of polylactic acid (PLA) in medical implants and theranostic systems. Chem. Eng. J..

[45] Van de Velde K., Kiekens P. (2002). Biopolymers: Overview of several properties and consequences on their applications. Polym. Test..

[46] Fiedler T., Belova I.V., Murch G.E., Roether J.A., Boccaccini A.R. (2012). Tailoring elastic properties of PLGA/TiO 2 biomaterials. Comput. Mater. Sci..

[47] Balla V.K., Bodhak S., Bose S., Bandyopadhyay A. (2010). Porous tantalum structures for bone implants: Fabrication, mechanical and in vitro biological properties. Acta Biomater..

[48] Matsuno H., Yokoyama A., Watari F., Uo M. (2001). Biocompatibility and osteogenesis of refractory metals. Biomaterials.

[49] Katona B., Szlancsik A., Tábi T., Orbulov I.N. (2019). Compressive characteristics and low frequency damping of aluminium matrix syntactic foams. Mater. Sci. Eng. A.

[50] Linul E., Marşavina L., Linul P.-A., Kovacik J. (2019). Cryogenic and high temperature compressive properties of Metal Foam Matrix Composites. Compos. Struct..

[51] Roy D.M., Linnehan S.K. (1974). Hydroxyapatite formed from coral skeletal carbonate by hydrothermal exchange. Nature.

[52] Leukers B., Gülkan H., Irsen S.H., Milz S., Tille C., Schieker M., Seitz H. (2005). Hydroxyapatite scaffolds for bone tissue engineering made by 3D printing. J. Mater. Sci. Mater. Med..

[53] Wu C., Fan W., Zhou Y., Luo Y., Gelinsky M., Chang J., Xiao Y. (2012). 3D-printing of highly uniform CaSiO3ceramic scaffolds: Preparation, characterization and in vivo osteogenesis. J. Mater. Chem..

[54] Basu B., Ghosh S. (2017). Biomaterials for Musculoskeletal Regeneration.

